# A rare entity of primary hydatid cyst located between the two layers of the intracranial dura in a child: a case report

**DOI:** 10.1093/omcr/omad107

**Published:** 2023-12-19

**Authors:** Hassan Kadri, Mazen Dughly, Raed Abouharb, Sameer Bakleh, Rostom Mackieh

**Affiliations:** Department of Neurosurgery, Children’s University Hospital, Faculty of Medicine, Damascus University, Damascus, Syria; Department of Neuroradiology Damascus National Hospital (DNH), Damascus, Syria; Department of Pediatrics, Children’s University Hospital, Faculty of Medicine, Damascus University, Damascus, Syria; Department of Pediatrics, Children’s University Hospital, Faculty of Medicine, Damascus University, Damascus, Syria; Department of Neurosurgery, Children’s University Hospital, Faculty of Medicine, Damascus University, Damascus, Syria

## Abstract

Introduction: Hydatid disease is a parasitic infection caused by the tapeworm *Echinococcus granulosus*. Intracranial locations are rare and account for less than 3% of all cases. Typically, these cysts are found in the intracerebral spaces. However, this study presents an extremely rare intradural hydatid cyst. To our knowledge no similar case has been previously reported. Case presentation: This study presents the case of an 8-year-old boy presented with a 3-month history of headache and vomiting without any neurological deficit. Full radiological investigations were performed, the brain MRI showed a large cerebral hydatid cyst located within the dura layers between the periosteal and the endosteal layers. Surgery was performed without cyst rupture, confirming the intracerebral intradural location. Conclusion: Early diagnosis and treatment for intracranial hydatid cysts are crucial to prevent complications such as neurological deficits, seizures, and even death. In this case, the intracerebral intradural location of the cyst is extremely rare.

## INTRODUCTION

Hydatid disease is a parasitic infection caused by the larval cysts of the tapeworm *Echinococcus granulosus*, which can form in various human tissues. The disease is acquired through the fecal-oral route, with humans being an incidental intermediate host. Central nervous system (CNS) involvement in hydatid disease is rare, with 2–3% of cases showing such involvement [1]. The disease can manifest as intracerebral and is more common in certain parts of the world, such as South America, Australia, the Middle East, and parts of North Africa [2].

In the northeast of Syria, hydatid cysts are relatively common, but most of these are found in the chest, liver, and abdomen. While hydatid disease has been eradicated in some countries, it remains a serious health problem in certain parts of the world, particularly in areas where livestock is raised close to humans [3]. Hydatid cysts can cause severe neurological symptoms, such as elevated intracranial pressure signs, seizures, and other neurological deficits, and require timely diagnosis and appropriate surgical management [4, 5].

## CASE REPORT

**Figure 1:**
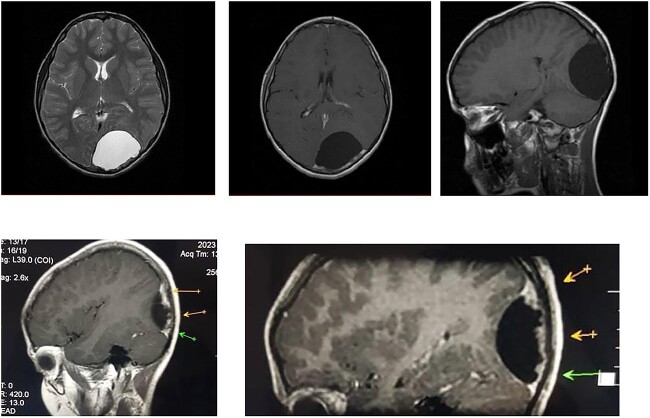
Brain MRI scan of the Hydatid Cyst. Meningeal enhancement around posterior margin of the cyst is seen suggestive for intradural location, the cyst is not exceeding the mid line as well.

We report the case of an 8-year-old male child who presented to the neurology department with complaints of headache and vomiting for 3 months. Physical examination did not reveal any focal neurological deficits. The patient was referred to neurosurgery and underwent brain magnetic resonance imaging (MRI) (Fig. 1). The imaging findings revealed a well-defined supratentorial cyst measuring approximately 4 × 5.5 × 6 cm located in the left occipital region. The cyst was isointense to CSF in all sequences, with faint isointensity in the fluid-attenuated inversion recovery (FLAIR) sequence, and was surrounded by a thin hypointense rim in T2* and T2WI. Minimal edema was noted anterior to the cyst, and there was no evidence of abnormal enhancement after contrast injection. However, the 3D thin slices showed meningeal enhancement around the upper margin of the lesion, suggesting that the cyst was located between meningeal layers. The posterior wall of the cyst appeared irregular and was associated with prominent enhancement at adjacent meninges, with irregular erosions of the adjacent occipital bone. The cyst was causing pressure effects on the straight and superior venous sinuses and the splenium of the corpus callosum and left lateral ventricle, and was deviating the midline to the right by approximately 8 mm. Radiological investigations of the chest and abdomen did not reveal any abnormalities.

**Figure 2:**
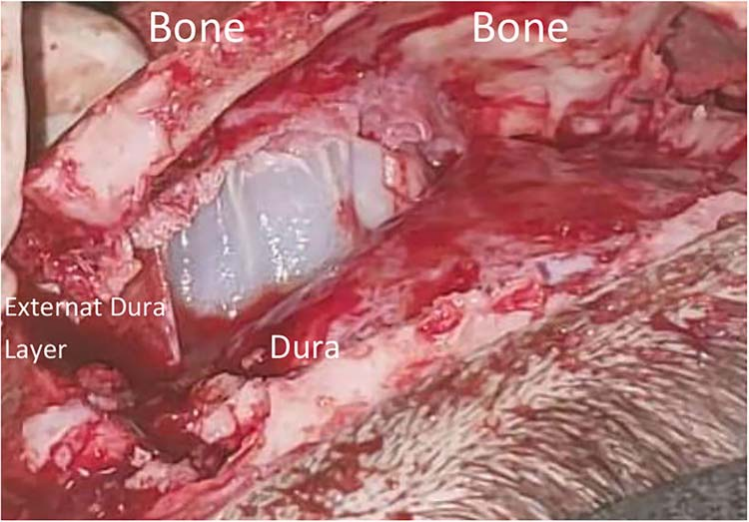
Intraoperative view of the cyst surrounded by the dura.

**Figure 3:**
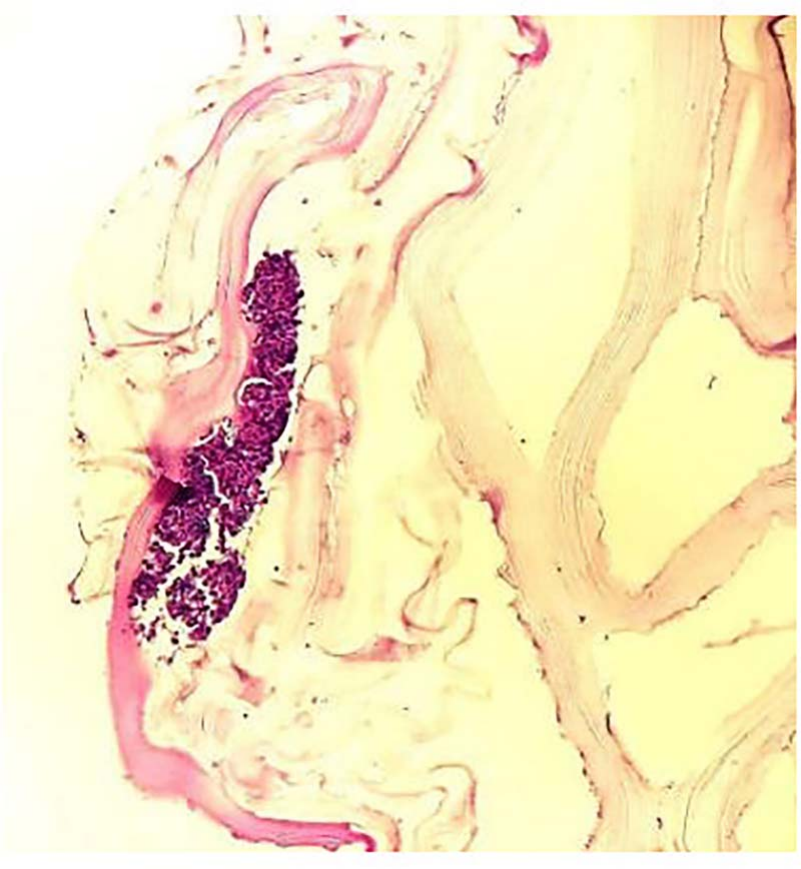
Hydatid cyst wall (40× magnification), showing cyst wall with three components, outer acellular lamellated membrane, germinal membrane and attached protoscolices. Increased eosinophils seen in the image.

The patient underwent a craniotomy (Fig. 2). While retracting the bone, a thin layer of periosteal layer of the dura covering a cyst was noticed. The dura was gently retracted, and the cyst was visualized. No rupture was noted, and the cyst was successfully removed, leaving the inner layer of the dura intact. This gentle maneuver during the craniotomy is essential to avoid any eventual rupture of any superficial cyst that could be located anywhere under the bone flap. Postoperatively patients had a completely normal neurological examination, he was referred to internal medicine for medical treatment by albendazole. The follow-up lasted for 3 months without any complications (Fig. 3).

## DISCUSSION

Here, we report a case of an 8-year-old boy from a northeast region of the country known for its endemicity of *E. granulosus*. He developed a hydatid cyst in an unusual location of the brain that was extracerebral intradural, which is an extremely rare occurrence.

Hydatid cysts, also known as *E. granulosus*, are parasitic infections that can affect various organs in the body, including the brain. Although less than 3% of hydatid cysts occur in the brain, they are predominantly seen as intracerebral [6]. However, rare cases of primary multiple intracranial extradural hydatid cysts have been reported [7, 8]. A location in the extradural space of the posterior fossa was also described [9], and the presence of the primary cyst in the extradural space was seen as associated with other pathologies like nephrotic syndrome [10]. Additionally, there have been reports of intracranial intraosseous hydatid cysts [11]. Only a few cases have been reported to occur in the spine, accounting for approximately 1% of all hydatid cysts [12, 13]. Rupture or infection of the cyst is a serious complication that requires special management [9, 10]. Although diagnosing hydatid cysts can be challenging, imaging studies, including computed tomography and MRI, are essential for diagnosis [14, 15].

By carful understanding the preoperative evaluation and manipulating the bone flap during surgery, any incidental rupture of the HC can be avoided.

## CONCLUSION

Intradural hydatid cysts of the brain are extremely rare but can cause serious neurological complications. Early diagnosis and prompt treatment are essential for a favorable outcome. A high index of suspicion is necessary for patients from endemic areas who present with neurological symptoms. The treatment of choice is complete surgical excision of the cyst, followed by albendazole therapy for 6 months. Surgeons should be aware of the cyst’s superficial location when removing the bone flap to avoid rupturing it. These cysts can be located under the external layer of the dura, which is very fragile. The outcome is usually good after surgical removal and medical treatment in the absence of any incidental surgical rupture.

## Data Availability

All data and medical information used in this study are available in the archives of the Children’s University Hospital in Damascus, Syria, and can be verified upon request.
